# Thrombolytic therapy for haemodialysis catheter dysfunction – value for money?

**DOI:** 10.1080/0886022X.2020.1788583

**Published:** 2020-07-08

**Authors:** Julian Yaxley, Jagadeesh Kurtkoti, Linda Stockwell

**Affiliations:** a Department of Nephrology, Gold Coast University Hospital, Gold Coast, Australia; b Griffith University School of Medicine, Gold Coast, Australia

Sir,

Many dialysis patients rely on tunneled central venous catheters for long-term vascular access. Hemodialysis catheter dysfunction (HCD) caused by intraluminal thrombosis is a common problem often necessitating replacement. We have noticed increasing prescription of thrombolytic therapy for HCD across our health service in recent years. Thrombolytic agents are relatively costly and evidence for their use and cost-effectiveness in treating HCD is sparse. Given budgetary considerations in our health service, we sought to examine this subject by auditing our hospital records. We intended to observe local practices regarding HCD and evaluate whether adherence to standard guidelines improves the efficacy of thrombolysis.

We performed a retrospective chart review of all patients receiving intra-catheter thrombolysis for tunneled HCD over 12 months in our hospital network from August 2018. Although HCD is variably defined, we defined it for this audit as documented extracorporeal blood flow less than 300 mL/min despite an arterial line pressure exceeding negative 200 mmHg. We considered successful treatment to be improvement in catheter function to below that threshold during the next dialysis session. This quality improvement project was permitted by our local research committee.

Thrombolysis was administered on 66 occasions to 30 different patients in the 12-month period, at a cumulative cost of AU$12,795.42. Intravenous alteplase is the only agent used for this purpose in our renal unit, dosed in vials of 2.5 mg/2.5 mL at a price of AU$193.87 per vial. Agents available at other centers may include urokinase, streptokinase, and tenecteplase. Thrombolysis successfully reversed HCD in 56% (36/64) of cases. The median catheter survival after administration of intraluminal thrombolysis was 40 days and censored 3-month catheter survival was 31%. Although some machine and flow parameters were documented on most occasions, objective measurements satisfying the criteria for HCD were present in only 44% (29/66) of patients, indicating that thrombolysis may have been prescribed inappropriately in a number of cases. In patients who met the criteria, based on documented readings, thrombolysis efficacy increased to 64% (18/28).

In addition to intra-catheter thrombolysis, other basic interventions for HCD can include evaluating fluid status to avoid hypovolaemia and obtaining a chest radiograph to confirm catheter tip position [1]. There is no standardized practice at our center. In our audit, few patients received these measures in the week prior to thrombolysis, at 39% (26/66) and 18% (12/66) respectively. Thrombolytic therapy was more successful in those in whom the steps were taken.

**Figure 1.**
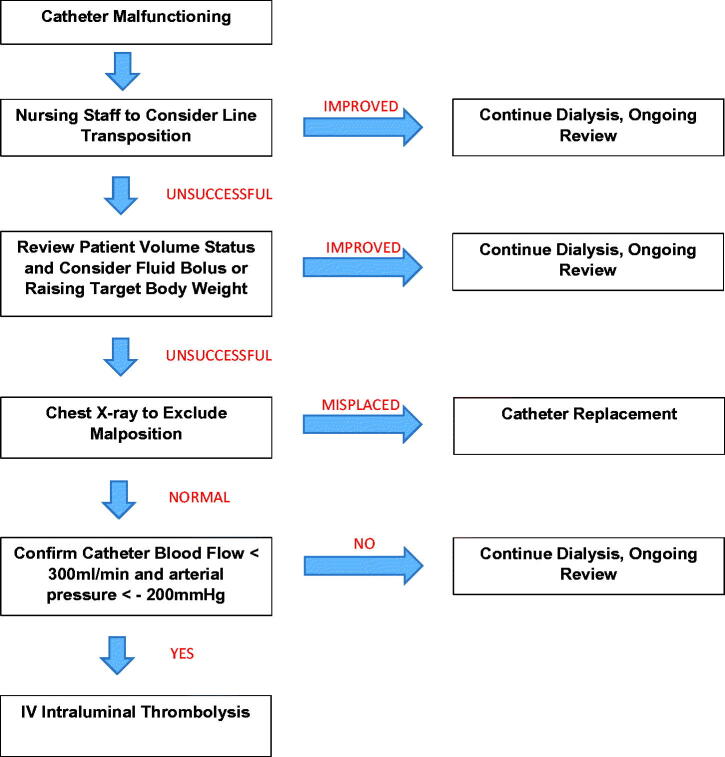
Flowchart of suggested approach to catheter dysfunction in our renal unit.

We presented our findings to our local renal unit and described an unofficial protocol (Figure 1) including simple interventions and adequate documentation prior to thrombolysis. We then prospectively audited the identical dataset for a further 6 months. In the subsequent 6-month period, a drastic reduction in thrombolytic therapy was observed. Alteplase was prescribed on 12 occasions which represents a greater than 50% reduction in use. The likelihood of chest X-ray, target weight modification, and objective quantification of HCD each increased by approximately 20% in the 6-month period. The overall success rate of thrombolysis in the second study period improved demonstrably to 75% (9/12) with a 3-month censored survival of 44%.

Intraluminal thrombosis accounts for approximately two thirds of catheter replacements [2]. Most tunneled hemodialysis central venous catheters do not last for more than 1 year and interventions to improve their longevity are poorly studied [2]. While there is probably no single best approach, in the authors’ experience most dialysis units do not have a formalized protocol for this issue and local practices vary widely. Within the limits of a chart review design, our findings suggest that a standardized approach may improve the utility of intra-catheter thrombolysis. Naturally, there are significant inherent weaknesses in an audit design and many confounding factors go unaccounted for.

Most of the interventions reviewed in our audit, to the author’s knowledge, have not been previously examined in the literature. The results of this chart review are encouraging and generate the hypothesis that the utility and cost-effectiveness of thrombolysis for HCD can be improved with judicious use. This is an important subject for nephrologists and warrants further high quality clinical research.
